# Frailty in hypertensive population and its association with all-cause mortality: data from the National Health and Nutrition Examination Survey

**DOI:** 10.3389/fcvm.2023.945468

**Published:** 2023-04-25

**Authors:** Li Li, Yuge Wang, Chunlei Yang, Chenhui Huang, Lanzhi Duan, Jianghua Zhou, Yanyu Lu, Guojun Zhao

**Affiliations:** ^1^ Department of Cardiovascular Surgery, The First Affliated Hospital of Zhengzhou University, Zhengzhou, China; ^2^ Department of Medicine, Jinggangshan University, Ji'an, China; ^3^ Department of Cardiology, The First Affliated Hospital of Zhengzhou University, Zhengzhou, China; ^4^ College of Nursing, Wenzhou Medical University, Wenzhou, China; ^5^ Department of Cardiology, The First Affliated Hospital of Wenzhou Medical University, Wenzhou, China

**Keywords:** frailty, pre-frailty, mortality, hypertension, NHANES

## Abstract

**Objectives:**

This study aimed to investigate the relationship between frailty and all-cause mortality in hypertensive population.

**Methods:**

We used data from the National Health and Nutrition Examination Survey (NHANES) 1999–2002 and mortality data from the National Death Index. Frailty was assessed using the revised version of the Fried frailty criteria (weakness, exhaustion, low physical activity, shrinking, and slowness). This study aimed to evaluate the association between frailty and all-cause mortality. Cox proportional hazard models were used to evaluate the association between frailty category and all-cause mortality, adjusted for age, sex, race, education, poverty–income ratio, smoking, alcohol, diabetes, arthritis, congestive heart failure, coronary heart disease, stroke, overweight, cancer or malignancy, chronic obstructive pulmonary disease, chronic kidney disease, and taking medicine for hypertension.

**Results:**

We gathered data of 2,117 participants with hypertension; 17.81%, 28.77%, and 53.42% were classified as frail, pre-frail, and robust, respectively. We found that frail [hazard ratio (HR) = 2.76, 95% confidence interval (CI) = 2.33–3.27] and pre-frail (HR = 1.38, 95% CI = 1.19–1.59] were significantly associated with all-cause mortality after controlling for variables. We found that frail (HR = 3.02, 95% CI = 2.50–3.65) and pre-frail (HR = 1.35, 95% CI = 1.15–1.58) were associated with all-cause mortality in the age group ≥65 years. For the frailty components, weakness (HR = 1.77, 95% CI = 1.55–2.03), exhaustion (HR = 2.25, 95% CI = 1.92–2.65), low physical activity (HR = 2.25, 95% CI = 1.95–2.61), shrinking (HR = 1.48, 95% CI = 1.13–1.92), and slowness (HR = 1.44, 95% CI = 1.22–1.69) were associated with all-cause mortality.

**Conclusion:**

This study demonstrated that frailty and pre-frailty were associated with an increased risk of all-cause mortality in patients with hypertension. More attention should be paid to frailty in hypertensive patients, and interventions to reduce the burden of frailty may improve outcomes in these patients.

## Introduction

Hypertension is becoming increasingly prevalent globally, prompting the development of cardiovascular disease. The number of hypertensive patients aged 30–79 years has rapidly increased from 331 million women and 317 million men in 1990 to 626 million women and 652 million men in 2019 (1). The increasing prevalence and incidence of hypertension have increased the burden of the disease. For instance, hypertension, pre-hypertension, and other hazardous high blood pressure conditions were responsible for approximately 8.5 million deaths in 2015 from ischemic heart disease, stroke, other vascular diseases, and renal disease (2).

Frailty is defined as reduced functioning of multiple physiological systems and increased vulnerability to stressors (3), which is associated with an increased risk of mortality, falls, disability, and higher healthcare costs (4–7). The prevalence of frailty is estimated to be 17.81%, while that of pre-frailty is 28.77% among older persons living in the community (8). Emerging evidence suggests that hypertension is associated with a higher prevalence of frailty. A recent meta-analysis of 23 cross-sectional studies demonstrated that the prevalence of frailty in individuals with hypertension is 14%. However, the relationship between frailty and all-cause mortality in individuals with hypertension remains unclear. Although frailty in community-dwelling hypertensive older Chinese adults was associated with increased risk of poorer physical function and higher mortality (9), data on the effects of pre-frailty and frailty on adverse outcomes, its sample size, and regional representation have been scarce. Therefore, this study aimed to assess the association between frailty and all-cause mortality in patients with hypertension from the National Health and Nutrition Survey (NHANES).

## Methods

### Study design and participants

**Figure 1:**
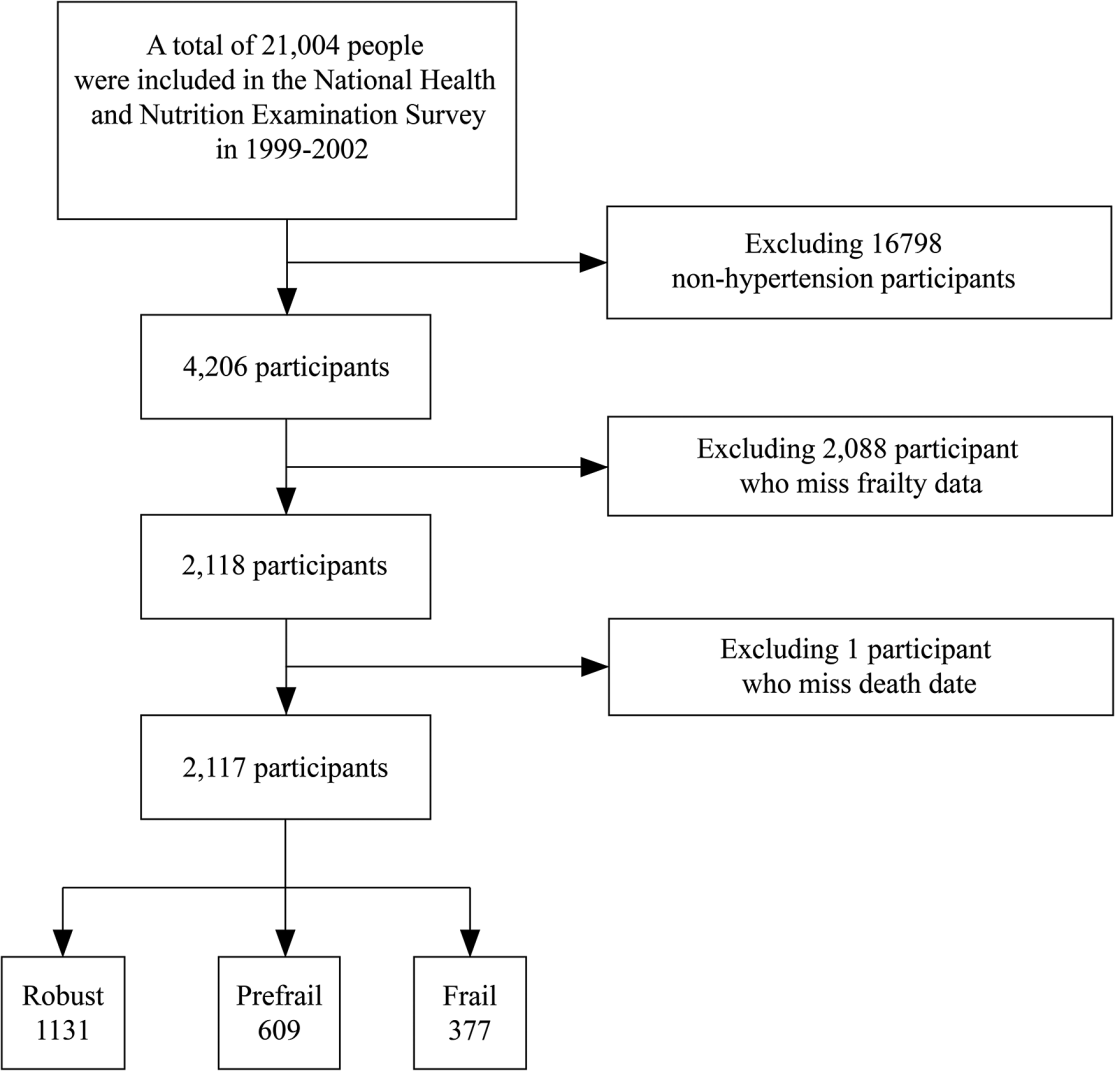
Flowchart of candidate selection in the current study.

This retrospective cohort study included participants from the NHANES 1999–2002. The NHANES is a multistage probabilistic survey conducted by the National Center for Health Statistics to understand and estimate the health and nutrition of adults and children in the United States. The key points of this survey were the inclusion of other Hispanics, non-Hispanic whites, non-Hispanic blacks, and adults aged ≥18 in Mexico and the United States. In total, 21,004 participants were included in the NHANES. We included only patients with hypertension (*n* = 4,206) who had full frailty and mortality data (*n* = 2,117) (Figure 1). Hypertension was defined as having a history of hypertension, raised blood pressure (systolic blood pressure (SBP) ≥ 140 and/or diastolic blood pressure (DBP) ≥ 90), or self-reported use of antihypertensive medication (10, 11). All participants provided informed consent and agreed to participate in this study.

### Mortality data

The mortality rate was assessed using the 2015 Public-use linked Mortality File, which covers mortality data from the study period to 31 December 2011. Data were obtained from the National Death Index, a special service of the National Center for Health Statistics, which is a centralized database of information on death records from the National Bureau of Vital Statistics. These data were linked to the NHANES data using a special study identifier. The time of death was calculated from the number of days since the date of death, and all-cause mortality was assessed.

### Study variables

We used data from participants’ self-reported questionnaires and objective measurements to apply the Fried definition of frailty to our study sample. We used five criteria of frailty (weakness, exhaustion, low physical activity, shrinkage, and slowness) that were also used in previous NHANES data (12). Weakness included answering “have difficulty,” “very difficult,” or “can't” when asked about difficulty lifting or carrying 10 pounds; exhaustion included reporting “some difficulty” or “very difficult” when asked about difficulty in walking between rooms; and low physical activity included when asked “Compared to most men/women your age, do you think you are more active, less active, or about the same?”; shrinking includes an unexpected weight loss of at least 10 pounds or at least 5% of BMI within a year; slowness included walking speed scores (20 ft test, usual pace, one tail), adjusted for gender and standing height (men: height ≤173 cm and speed ≤0.6531 m/s; height >173 cm and speed ≤0.762 m/s; women: height ≤159 cm and speed ≤0 6,531 m/s; height >159 cm and speed ≤0.762 m/s) (Supplementary Table S1). Frail, pre-frail, and robust were defined as meeting three or more, one or two, or zero of the five criteria, respectively.

### Covariates

The demographic variables included self-reported age, sex, race, education, poverty–income ratio, smoking, and alcohol. We divided the respondents into Mexican Americans, other Hispanics, non-Hispanic whites, non-Hispanic blacks, and others. If respondents answered the question “Has a doctor told you have a disease state?”, we could identify self-reported comorbidities such as diabetes, arthritis, congestive heart failure, coronary artery disease, stroke, overweight, and cancer or malignancy. Patients with chronic obstructive pulmonary disease (COPD) and chronic kidney disease (CKD) were not included in the NHANES database. COPD was defined as a positive response to either chronic bronchitis or emphysema, and a negative response to current asthma (13). CKD was defined as an estimated glomerular filtration rate <60 ml/min/1.73 m^2^ and/or a urinary albumin–creatinine ratio (ACR) >30 mg/g (14). Participants were classified as smokers if they had smoked at least 100 cigarettes during their lifetime. Taking medicine for hypertension was listed as no (means participants did not take any medicine for hypertension) or yes (means participants taking one or more medicines for hypertension).

### Statistical analysis

All the data were combined into a single file for systematic analysis. Continuous variables are expressed as mean and SE, while classified variables are expressed as weighted percentages and counts. Chi-square test and ANOVA were used to assess differences in baseline characteristics in the frailty group. Because some covariables had missing values, we explain the missing values using multiple imputation analyses. Multiple imputation was performed using R version 3.4.3 and multivariate imputation by chained equation (MICE) package by creating reasonable data values in a specially designed distribution for each data point. We generated five interpolation datasets using the random forest method. Adjusted variables included education, poverty–income ratio, diabetes, arthritis, congestive heart failure, coronary heart disease, stroke, overweight, cancer or malignancy, COPD, CKD, and taking medicine for hypertension. Five datasets were averaged to obtain the final imputation data set for the analysis. To test the quality of interpolation, we analyzed the subset excluding interpolation variables and the complete interpolation data set.

**Figure 2:**
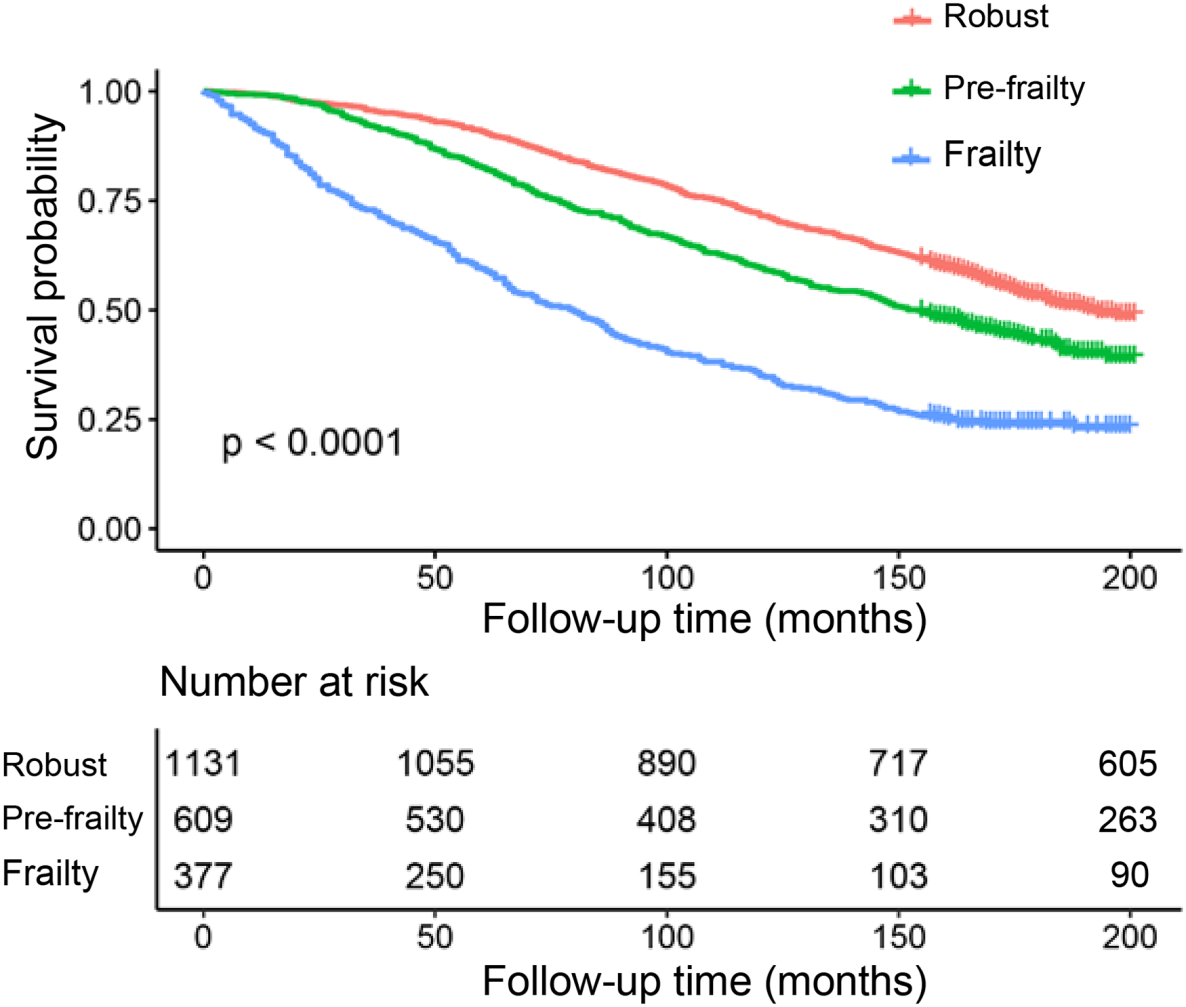
The Kaplan-Meier survival curves for all-cause mortality.

Mortality risk and frailty category were the primary outcome and predictor, respectively. Three separate Cox proportional risk models were developed to assess the mortality risk. Model 1 was unadjusted, with frailty category as the only predictor. Model 2 was adjusted for sex, race, education level, and poverty–income ratio. Model 3 contained the covariates of Model 2, and was additionally adjusted for smoking, alcohol, diabetes, arthritis, congestive heart failure, coronary heart disease, stroke, overweight, cancer or malignancy, COPD, CKD, and taking medicine for hypertension. All-cause mortality was assessed, and the results were expressed as hazard ratios (HRs) with 95% confidence intervals (CIs). The Kaplan–Meier survival curves for all-cause mortality are shown in Figure 2. As an exploratory analysis, the model was stratified according to age groups (<65 and ≥65 years). Statistical software R (version 3.4.3) and Empower (R) (http://www.empowerstats.net/cn/index.php) were used to analyze the data.

## Results

The basic characteristics of frail, pre-frail, and robust are shown in Table 1. All-cause mortality and frailty were assessed in 2,117 participants with hypertension. The participants’ average age was 70.03 ± 10.14 years. Participants’ characteristics differed in frail, pre-frail, and robust, including poverty–income ratio, race, sex, education, alcohol consumption, diabetes, arthritis, congestive heart failure, coronary heart disease, stroke, overweight, COPD, and CKD.

**Table 1:** Baseline characteristics of the patients with different frailty status.

Characteristics	Total (*N* = 2,117)	Robust (*N* = 1,131)	Pre-frail (*N* = 609)	Frail (*N* = 377)	*P*-value
Age, mean (SE)	70.03 (10.14)	70.21 (8.54)	69.96 (10.59)	69.59 (13.33)	0.277
Poverty–income ratio, *n* (%)	2.43 (1.48)	2.77 (1.47)	2.22 (1.45)	1.75 (1.24)	<0.001
Age, *n* (%)					0.733
<65	605 (28.58%)	319 (28.21%)	172 (28.24%)	114 (30.24%)	
≥65	1,512 (71.42%)	812 (71.79%)	437 (71.76%)	263 (69.76%)	
Sex, *n* (%)					<0.001
Male	1,005 (47.47%)	597 (52.79%)	269 (44.17%)	139 (36.87%)	
Female	1,112 (52.53%)	534 (47.21%)	340 (55.83%)	238 (63.13%)	
Race, *n* (%)					<0.001
Mexican American	388 (18.33%)	194 (17.15%)	120 (19.70%)	74 (19.63%)	
Other Hispanic	90 (4.25%)	44 (3.89%)	24 (3.94%)	22 (5.84%)	
Non-Hispanic white	1,154 (54.51%)	671 (59.33%)	313 (51.40%)	170 (45.09%)	
Non-Hispanic black	442 (20.88%)	202 (17.86%)	139 (22.82%)	101 (26.79%)	
Other	43 (2.03%)	20 (1.77%)	13 (2.13%)	10 (2.65%)	
Education, *n* (%)					<0.001
Less than high school	904 (42.70%)	381 (33.69%)	306 (50.25%)	217 (57.56%)	
High school diploma	500 (23.62%)	290 (25.64%)	136 (22.33%)	74 (19.63%)	
More than high school	713 (33.68%)	460 (40.67%)	167 (27.42%)	86 (22.81%)	
Smoking, mean (SE)	1,097 (51.82%)	574 (50.75%)	321 (52.71%)	202 (53.58%)	0.555
Alcohol, mean (SE)	1,264 (59.71%)	718 (63.48%)	348 (57.14%)	198 (52.52%)	<0.001
Comorbidities, *n* (%)					
Diabetes	453 (21.40%)	183 (16.18%)	126 (20.69%)	144 (38.20%)	<0.001
Arthritis	1,024 (48.37%)	453 (40.05%)	323 (53.04%)	248 (65.78%)	<0.001
Congestive heart failure	181 (8.55%)	40 (3.54%)	61 (10.02%)	80 (21.22%)	<0.001
Coronary heart disease	238 (11.24%)	94 (8.31%)	76 (12.48%)	68 (18.04%)	<0.001
Stroke	210 (9.92%)	70 (6.19%)	45 (7.39%)	95 (25.20%)	<0.001
Overweight	856 (40.43%)	393 (34.75%)	274 (44.99%)	189 (50.13%)	<0.001
Cancer or malignancy	377 (17.81%)	204 (18.04%)	101 (16.58%)	72 (19.10%)	0.579
COPD	143 (6.75%)	57 (5.04%)	40 (6.57%)	46 (12.20%)	<0.001
CKD	840 (39.68%)	355 (31.39%)	270 (44.33%)	215 (57.03%)	<0.001
Take medicine for hypertension					0.567
Yes	426 (20.12%)	232 (20.51%)	114 (18.72%)	80 (21.22%)	
No	1,691 (79.88%)	899 (79.49%)	495 (81.28%)	297 (78.78%)	

COPD, chronic obstructive pulmonary disease; CKD, chronic kidney disease.

Supplementary Table S2 presents the classification of each frailty component, the number of components participants in the study fulfilled, and overall rates of participants who were frail (53.42%), pre-frail (28.77%), and robust (17.81%). The frailty component with the highest rate was weakness (31.6%), followed by low physical activity (22.7%). Very few participants met all five criteria (<1%).

Multivariate logistic regression analysis of predictors of frailty (Table 2) revealed that female sex, high school diploma, poverty–income ratio, diabetes, arthritis, congestive heart failure, stroke, overweight, and CKD were predictors of frailty.

**Table 2:** Multivariate logistic analysis of predictors for frailty.

	Pre-frail		Frail	
	Odds ratio (95% CI)	*P*-value	Odds ratio (95% CI)	*P*-value
Sex (female)	1.44 (1.14–1.82)	<0.001	2.65 (1.89–3.70)	<0.001
Education				
High school diploma	0.64 (0.48–0.84)	0.001	0.54 (0.37–0.79)	0.001
More than high school	0.63 (0.48–0.83)	0.001	0.84 (0.58–1.23)	0.380
Poverty–income ratio	0.83 (0.76–0.90)	<0.001	0.64 (0.57–0.72)	<0.001
Alcohol	1.02 (0.89–1.29)	0.870	1.20 (0.68–1.66)	0.270
Comorbidities				
Diabetes	0.88 (0.66–1.16)	0.360	1.58 (1.12–2.22)	0.010
Arthritis	1.44 (1.16–1.78)	0.001	1.93 (1.42–2.63)	<0.001
Congestive heart failure	2.04 (1.28–3.25)	0.003	3.06 (1.80–5.20)	<0.001
Coronary heart disease	1.43 (0.98–2.07)	0.060	1.43 (0.87–2.33)	0.160
Stroke	0.90 (0.59–1.36)	0.610	2.99 (1.94–4.58)	<0.001
Overweight	1.60 (1.23–2.09)	<0.001	1.57 (1.09–2.27)	0.020
Cancer or malignancy	0.81 (0.52–1.29)	0.385	1.20 (0.64–2.26)	0.571
COPD	1.05 (0.67–1.65)	0.840	1.37 (0.80–2.34)	0.240
CKD	1.54 (1.24–1.93)	<0.001	1.87 (1.39–2.53)	<0.001
Take medicine for hypertension	0.93 (0.71–1.21)	0.572	1.17 (0.82–1.67)	0.399

COPD, chronic obstructive pulmonary disease; CKD, chronic kidney disease; CI, confidence interval.

Using stratified analysis, we found that sex, age, Mexican American, non-Hispanic white, non-Hispanic black, education, diabetes, arthritis, congestive heart failure, stroke, overweight, cancer or malignancy, CKD, and taking medicine for hypertension were significantly different between frailty and all-cause mortality. In addition, age was found to be an interaction factor in the interaction test (Supplementary Table S3).

Cox regression analyses of all-cause mortality among participants with frailty (Table 3) showed that frail (HR = 2.76, 95% CI = 2.33–3.27) and pre-frail (HR = 1.38, 95% CI = 1.19–1.59) were significantly associated with all-cause mortality, after controlling for variables. We found that frail (HR = 3.02, 95% CI = 2.50–3.65) and pre-frail (HR = 1.35, 95% CI = 1.15–1.58) were associated with all-cause mortality in the age group ≥65 years, frail (HR = 2.15, 95%CI = 1.43–3.25) and pre-frail (HR = 1.62, 95%CI = 1.14–2.32) were associated with all-cause mortality in the age group <65 years. For the frailty components, weakness (HR = 1.77, 95% CI = 1.55–2.03), exhaustion (HR = 2.25, 95% CI = 1.92–2.65), low physical activity (HR = 2.25, 95% CI = 1.95–2.61), shrinking (HR = 1.48, 95% CI = 1.13–1.92), and slowness (HR = 1.44, 95% CI = 1.22–1.69) were associated with all-cause mortality.

**Table 3:** Cox regression analyses of all-cause mortality among participants with frailty.

Mortality	Model I	Model II	Model III
Age < 65 years			
Robust	Ref	Ref	Ref
Pre-frail	1.89 (1.35–2.65) <0.001	1.77 (1.25–2.49) 0.001	1.62 (1.14–2.32) 0.008
Frail	2.89 (2.03–4.12) <0.001	2.60 (1.77–3.81) <0.001	2.15 (1.43–3.25) <0.001
*P* for trend	<0.001	<0.001	0.001
Age ≥ 65 years			
Robust	Ref	Ref	Ref
Pre-frail	1.33 (1.15–1.55) <0.001	1.43 (1.23–1.67) <0.001	1.35 (1.15–1.58) <0.001
Frail	3.30 (2.81–3.87) <0.001	3.65 (3.09–4.33) <0.001	3.02 (2.50–3.65) <0.001
*P* for trend	<0.001	<0.001	<0.001
Total			
Robust	Ref	Ref	Ref
Pre-frail	1.41 (1.23–1.61) <0.001	1.46 (1.27–1.68) <0.001	1.38 (1.19–1.59) <0.001
Frail	3.19 (2.76–3.69) <0.001	3.39 (2.90–3.96) <0.001	2.76 (2.33–3.27) <0.001
*P* for trend	<0.001	<0.001	<0.001
Weakness	1.74 (1.54–1.97) <0.001	2.06 (1.82–2.34) <0.001	1.77 (1.55–2.03) <0.001
Exhaustion	2.43 (2.10–2.80) <0.001	2.83 (2.44–3.29) <0.001	2.25 (1.92–2.65) <0.001
Low physical activity	1.76 (1.54–2.01) <0.001	2.63 (2.29–3.01) <0.001	2.25 (1.95–2.61) <0.001
Shrinking	2.40 (1.90–3.02) <0.001	1.68 (1.31–2.15) <0.001	1.48 (1.13–1.92) 0.003
Slowness	2.12 (1.83–2.46) <0.001	1.62 (1.38–1.89) <0.001	1.44 (1.22–1.69) <0.001

Model 1: unadjusted; Model 2: adjusted for sex, race, education, poverty–income ratio; Model 3: adjusted for Model 2 covariates and smoking, alcohol, diabetes, arthritis, congestive heart failure, coronary heart disease, stroke, overweight, cancer or malignancy, COPD, CKD, and taking medicine for hypertension.

Figure 2 shows the Kaplan–Meier survival curves for all-cause mortality, which showed that hypertension with a higher frailty state had a higher all-cause mortality. During follow-up, 1,153 participants died. The mean follow-up time was 130.1 months.

## Discussion

We found that frailty and pre-frailty were associated with an increased risk of death in individuals with hypertension. We assessed the association between frailty and mortality in 2,117 patients with hypertension. This study showed that frailty and pre-frailty in hypertensive patients increase the risk of mortality and are associated with both older and younger adults. In particular, pre-frail and frail individuals were 29% and 133% more likely to die than robust individuals, respectively. Determining this association is important for clarifying the advantages of being an important diagnostic entity vs. another early disease state.

Although frailty was widely regarded as a predictor of mortality in patients with heart failure (15, 16), few studies evaluated the relationship between frailty and mortality in hypertensive patients. A study of frailty, and the prevalence, associated effects, and prediction of long-term mortality in patients with hypertension, demonstrated that frailty was associated with a higher 8-year mortality (9), but the study included Chinese people, not Americans or other nations globally. Another study with an elderly Korean population demonstrated that among elderly patients with hypertension, frailty was more likely to be treated than pre-frailty or robustness (17). Moreover, frailty has been demonstrated to be associated with a worse quality of life, increased risk of all-cause hospitalization, and injurious falls in hypertensive adults (18, 19). Therefore, more attention should be paid to frailty in patients with hypertension, and interventions to reduce the burden of frailty may improve outcomes in these patients.

The potential mechanism for frailty with worse outcomes in patients with hypertension can be explained in several ways. First, frailty was demonstrated to be associated with limited physical activity and lower activities of daily living scores in patients with hypertension (20). Patients with impaired ambulation and disability levels, such as abnormal timed up-and-go test, may show a higher incidence of myocardial infarction and stroke, which is associated with an increased risk of death in patients (21, 22). Second, frail patients often have more physical and mental complications than non-frail patients do. Wong CH et al. demonstrated that 67.8% of patients had frailty and comorbidities (23), which may increase the vulnerability to adverse outcomes in frail patients under the relative stress level (24). Third, frailty is associated with higher levels of inflammation and oxidative stress; these mechanisms potentially exacerbate the cardiovascular status of hypertensive patients and increase the risk of cardiovascular events (25, 26).

Five criteria were used for frailty in this study, and 17.81% of patients were diagnosed with frailty. Results from a previous meta-analysis demonstrated that 14% of hypertensive patients had frailty (27). A higher prevalence of frailty redirects more attention to the risk factors of frailty in hypertensive patients, which we investigated in this study. Comorbidities, including diabetes, arthritis, congestive heart failure, stroke, overweight, and CKD were strong predictors of frailty in hypertensive patients. This indicates that multidisciplinary professional teams in the management of diseases, rehabilitation, nutrition, and psychology will be more helpful for hypertensive patients with frailty.

Moreover, the present study is the first to investigate the association between five frailty parameters and adverse clinical outcomes in hypertensive patients. We found that all frailty parameters were predictors of all-cause mortality in hypertensive patients. This is consistent with the results of previous studies. A meta-analysis of 48 independent cohorts reported that slow gait speed was associated with a 12% increased risk of all-cause mortality, and an 8% increased risk of chronic vascular disease (CVD) in elderly adults (28). In addition, low physical activity is significantly associated with shortened telomere length and increased mortality risk (29).

The present study has several limitations. First, the definition of frailty in our study was entirely based on the Fried frailty model; a number of the frailty components are based on self-reported data and are subject to recall bias. In addition, the Fried frailty model lacked cognitive and psychosocial factors to determine frailty. However, by reviewing a large body of literature, we found the Fried's definition of frailty was also used in other studies with data from NHANES (4, 30–33). Therefore, further studies are needed to assess the effects of frailty on the outcomes of hypertensive patients with more frailty criteria. Second, our covariable was absent, which may have weakened the reliability of our results. Fortunately, we interpreted the missing values through multiple attribution analyses and performed multiple imputations by creating reasonable data values for each data point in a specially designed distribution, which is a robust method that generates multiple predictions for each missing value (4). Third, we did not obtain multiple time monitoring of frailty during follow-up, which may have evaluated the association of frailty trajectory with clinical outcomes in patients with hypertension.

## Conclusions

In summary, this study demonstrated that frailty and pre-frailty were associated with an increased risk of all-cause mortality in patients with hypertension. More attention should be paid to frailty in hypertensive patients, and interventions to reduce frailty may improve outcomes in these patients.

## Data Availability

The original contributions presented in the study are included in the article/Supplementary Material, further inquiries can be directed to the corresponding author.
