# Supplementary material for: Frailty in hypertensive population and its association with all-cause mortality: data from the National Health and Nutrition Examination Survey
Source: Front Cardiovasc Med. 2023 Apr 25;10:945468. doi: 10.3389/fcvm.2023.945468 (PMC10167047; doi:10.3389/fcvm.2023.945468)
Supplement: Supplementary file 1 [file Datasheet1.docx]

**Table S1 Frailty criteria**

| criteria |  |
| --- | --- |
| Weakness | Reporting “some difficulty,” “much difficulty,” or “un able to do” when asked about difficulty lifting or carrying some thing as heavy as 10 pounds |
| Exhaustion | Reporting “some difficulty” or “much difficulty” when asked about difficulty walking between rooms. |
| Low physical activity | Reporting “less active” when asked “com pared with most men/women your age, would you say that you are more active, less active, or about the same.” |
| Shrinking | Unintentional weight loss of at least 10 lb or at least 5% over 1 year or Body mass index <18.5 kg/m2. |
| Slowness | Walking speed score (20ft test, usual pace, one tial),adjusted for sex and standing height |
|  | 一Men: height ≤173 cm and speed ≤0.6531 m/s; height > 173 cm and speed ≤0.762 m/s |
|  | 一Women: height≤ 159 cm and speed≤0 6531 m/s; height>159cm and speed ≤0.762m/s |

**Table S2 Frailty Components and rates**

| **Frailty Components** | **n (weighted %)** |
| --- | --- |
| Weakness | 607(31.60) |
| Exhaustion | 319(16.61) |
| Low physical activity | 436(22.70) |
| Shrinking | 243(12.65) |
| Slowness | 31616.44) |
| Number of components |  |
| 0 | 1131(53.42) |
| 1 | 539(25.46) |
| 2 | 70(3.31) |
| 3 | 277(13.09) |
| 4 | 89(4.20) |
| 5 | 11(0.52) |
| Frailty status |  |
| Robust | 1131(53.42) |
| Pre-frail | 609(28.77) |
| Frail | 337(17.81) |

**Table S3** **The association between frailty and mortality among all/subgroup participants**

|  | Pre-frail | | Frail | |  |
| --- | --- | --- | --- | --- | --- |
|  | HR (95% CI) | P | HR (95% CI) | P | P for trend |
| Sex |  |  |  |  | 0.157 |
| Male | 1.25 (1.02, 1.53) | 0.030 | 1.69 (1.27, 2.24) | <0.001 |  |
| Female | 1.35 (1.09, 1.67) | 0.006 | 2.87 (2.25, 3.67) | <0.001 |  |
| Age |  |  |  |  | 0.042 |
| <65 | 1.55 (1.07, 2.24) | 0.019 | 2.00 (1.28, 3.13) | 0.002 |  |
| ≥65 | 1.27 (1.08, 1.49) | 0.003 | 2.50 (2.05, 3.05) | <0.001 |  |
| Race |  |  |  |  | 0.633 |
| Mexican American | 1.19 (0.82, 1.73) | 0.356 | 1.96 (1.24, 3.11) | 0.004 |  |
| Other Hispanic | 1.70 (0.57, 5.09) | 0.344 | 2.20 (0.58, 8.27) | 0.245 |  |
| Non-Hispanicwhite | 1.36 (1.13, 1.65) | 0.001 | 2.51 (1.95, 3.23) | <0.001 |  |
| Non-Hispanic black | 1.37 (0.98, 1.92) | 0.064 | 2.88 (1.97, 4.21) | <0.001 |  |
| Other | 2.50 (0.52, 11.96) | 0.251 | 1.00 (0.15, 6.67) | 1.000 |  |
| Education |  |  |  |  | 0.371 |
| Less than high school | 1.16 (0.93, 1.44) | 0.179 | 2.12 (1.65, 2.71) | <0.001 |  |
| High school diploma | 1.60 (1.18, 2.17) | 0.002 | 3.20 (2.14, 4.77) | <0.001 |  |
| More than high school | 1.36 (1.04, 1.78) | 0.026 | 2.31 (1.58, 3.39) | <0.001 |  |
| Diabetes |  |  |  |  | 0.887 |
| Yes | 1.20 (0.88, 1.65) | 0.254 | 2.00 (1.44, 2.79) | <0.001 |  |
| No | 1.34 (1.14, 1.58) | 0.001 | 2.50 (2.01, 3.12) | <0.001 |  |
| Arthritis |  |  |  |  | 0.062 |
| Yes | 1.20 (0.98, 1.49) | 0.083 | 2.54 (2.01, 3.22) | <0.001 |  |
| No | 1.41 (1.15, 1.72) | 0.001 | 1.92 (1.43, 2.58) | <0.001 |  |
| Congestive heart failure |  |  |  |  | 0.174 |
| Yes | 1.07 (0.64, 1.78) | 0.794 | 1.38 (0.81, 2.37) | 0.240 |  |
| No | 1.30 (1.12, 1.51) | 0.001 | 2.63 (2.17, 3.19) | <0.001 |  |
| Coronary heart disease |  |  |  |  | <0.001 |
| Yes | 1.39 (0.73, 2.66) | 0.320 | 2.64 (1.25, 5.61) | 0.011 |  |
| No | 1.48 (1.20, 1.83) | <0.001 | 3.63 (2.73, 4.82) | <0.001 |  |
| Stroke |  |  |  |  | 0.598 |
| Yes | 1.33 (0.82, 2.17) | 0.242 | 2.63 (1.61, 4.30) | <0.001 |  |
| No | 1.31 (1.13, 1.53) | 0.001 | 2.31 (1.90, 2.82) | <0.001 |  |
| Overweight |  |  |  |  | 0.847 |
| Yes | 1.18 (0.91, 1.52) | 0.212 | 2.25 (1.68, 3.01) | <0.001 |  |
| No | 1.34 (1.12, 1.61) | 0.001 | 2.28 (1.80, 2.89) | <0.001 |  |
| Cancer or malignancy |  |  |  |  | 0.536 |
| Yes | 1.22 (0.87, 1.70) | 0.245 | 2.06 (1.36, 3.13) | <0.001 |  |
| No | 1.31 (1.11, 1.54) | 0.001 | 2.35 (1.92, 2.87) | <0.001 |  |
| COPD |  |  |  |  | 0.875 |
| Yes | 0.95 (0.50, 1.80) | 0.867 | 1.75 (0.91, 3.37) | 0.092 |  |
| No | 1.32 (1.14, 1.53) | <0.001 | 2.35 (1.95, 2.84) | <0.001 |  |
| CKD |  |  |  |  | 0.057 |
| Yes | 1.23 (1.00, 1.50) | 0.050 | 2.39 (1.90, 3.01) | <0.001 |  |
| No | 1.38 (1.12, 1.71) | 0.002 | 2.08 (1.53, 2.82) | <0.001 |  |
| Take medicine for hypertension |  |  |  |  | 0.726 |
| Yes | 1.79 (1.13, 2.81) | 0.012 | 3.76 (2.13, 6.63) | <0.001 |  |
| No | 1.45 (1.17, 1.81) | 0.001 | 3.65 (2.71, 4.92) | <0.001 |  |
